# Comparison of the Immunogenicity and Safety of Two Pediatric TBE Vaccines Based on the Far Eastern and European Virus Subtypes

**DOI:** 10.1155/2019/5323428

**Published:** 2019-12-24

**Authors:** Mikhail F. Vorovitch, Galina B. Maikova, Liubov L. Chernokhaeva, Victor V. Romanenko, Galina G. Karganova, Aydar A. Ishmukhametov

**Affiliations:** ^1^Federal State Budgetary Scientific Institution “Chumakov Federal Scientific Center for Research and Development of Immune-and-biological Products of Russian Academy of Sciences” (FSBSI “Chumakov FSC R&D IBP RAS”), 108819 Moscow, Russia; ^2^Sechenov First Moscow State Medical University, 119991 Moscow, Russia; ^3^Federal Budgetary Healthcare Institution “Center for Hygiene and Epidemiology in the Sverdlovsk Region”, 620078 Yekaterinburg, Russia

## Abstract

Up to 10,000 cases of tick-borne encephalitis are registered annually, 20% of which occur in children under 17 years of age. A comparison of the immunogenicity and safety between a new pediatric Tick-E-Vac vaccine based on the TBEV strain Sofjin and FSME-IMMUN Junior vaccine was performed in the Sverdlovsk region. The vaccine strains differ from strains of the Siberian subtype of TBEV that dominates in the region. The study was performed on 163 children aged 1 to 15, who received one of the vaccines according to either a conventional or rapid vaccination schedule. Immunogenicity was assessed based on the seroprotection rates and titers of virus-neutralizing antibodies. There were no significant differences in either the immunogenicity or reactogenicity of the pediatric vaccines based on strains of the Far Eastern or European subtypes of TBEV. Under both vaccination schedules, 30 days after the second injection, seroprotection rates were 100% for Tick-E-Vac and greater than 95% for FSME-IMMUN Junior, while the geometric mean titer of TBEV-neutralizing antibodies was at least 2,4 log_10_ (1 : 250) for either vaccine. Fourteen days after the second injection according to the rapid schedule, seroprotection rates were significantly lower, ranging from 50% to 63% regardless of the vaccine used. The observed adverse reactions were mild or moderate for both vaccines under both vaccination schedules, with total adverse event rates of less than 25%. Reactogenicity was not associated with the gender or age of the recipients. There were no statistically significant differences in the incidence of adverse reactions between the group of subjects who were baseline seronegative or seropositive. However, 14 days after the second vaccine injection according to the rapid schedule, a statistically significant difference in nAbs titers was identified between groups of children with and without reported reactions.

## 1. Introduction

Tick-borne encephalitis (TBE) is a widespread infection in Central, Northern, and Eastern Europe, the Russian Federation, northern China, Mongolia, and Japan, with approximately 10,000 cases reported annually in the past decade [1]. More than 61 million people live in TBE-endemic regions of the Russian Federation [2], and at present, approximately 3,000 cases of this disease are registered each year. The geographic distribution of TBE is constantly increasing, and the incidence observed in children younger than 17 years has been rising, currently reaching 20% of the total number of cases [3]. It should also be noted that the prevalence of this infection has increased significantly in the pediatric population with ages under 3 years.

TBE is caused by the tick-borne encephalitis virus (TBEV), a member of the *Flavivirus* genus. Three main subtypes of TBEV were phylogenetically described and have been designated as the Far Eastern (FE), European (Eur), and Siberian (Sib) subtypes [4]. In Russia, all subtypes of the virus circulate, although the Sib subtype dominates [5].

Prevention of TBE through vaccination is the primary means of countering the disease. In the last two decades, considerable attention has been paid to TBE vaccine reactogenicity owing to a significantly increased number of vaccinated individuals, as well as a changing group composition and an expansion of the groups being vaccinated against TBE (e.g., children older than 1 year and elderly individuals) [6, 7].

The most widely used TBE vaccine in Russia is licensed for people 3 years old and older and is the universal lyophilized “TBE vaccine Moscow” vaccine, which is based on the Sofjin strain of the FE subtype [1, 8]. The primary vaccination course for this vaccine consists of only two injections within a 1- to 7-month interval. According to the results of our previous study using an animal model, this vaccine provides a wide range of protective immunity against all subtypes of TBEV [9]. However, the domestic commercial TBE vaccine intended for use in young children (aged 1 to 3 years) was not available in Russia until 2012, and imported vaccines based on the Eur strains of TBEV were used for childhood immunization.

A new liquid adsorbed TBE vaccine (Tick-E-Vac) based on the FE strain Sofjin was developed in Chumakov Federal Scientific Center. The vaccine contains purified formaldehyde-inactivated virions, adsorbed on aluminum hydroxide [1]. The pediatric form of Tick-E-Vac was designed to protect children aged 1–15 years with a recommended dose of 0.25 ml. A clinical study using the pediatric form of Tick-E-Vac was performed in the Sverdlovsk region, where the Siberian subtype of TBEV dominates [10]. The FSME-IMMUN Junior vaccine based on the Eur strain Neudoerfl and known for its low adverse event rates and high immunogenicity [7] was chosen as a comparison.

The main objective of this work was to evaluate and compare the immunogenicity and reactogenicity of the vaccines based on the FE and Eur strains in children aged 1–15 years after vaccination according to two different immunization schedules—conventional and rapid. The data from comparative studies in children are still somewhat limited.

## 2. Materials and Methods

### 2.1. Vaccines

Tick-E-Vac (dose 0.25 ml, batch No. 6.2009) contains purified formaldehyde-inactivated virions of the FE strain Sofjin, adsorbed on aluminum hydroxide. In brief, TBEV was propagated in primary chicken embryo fibroblast culture, inactivated, concentrated, and purified by exclusion gel chromatography. According to the manufacturer's data, the protein E content in a vaccine dose is 0.45 ± 0.05 *μ*g. The commercially available FSME-IMMUN Junior vaccine based on the Eur strain Neudoerfl (dose 0.25 ml, in a prefilled syringe, batch VNR1J08D) manufactured by Baxter Vaccine AG, Austria, was used as a comparison drug. According to the manufacturer's instruction, the protein E content in a vaccine dose is 1.19 *μ*g.

### 2.2. Vaccination

The reactogenicity and immunogenicity of the study vaccines were evaluated using the results of clinical trials conducted in the municipal institution “Children's Hospital No. 11” in Yekaterinburg, Russia. The study was monocentric, randomized, comparative, double-blind, controlled and performed in accordance with ICH-GCP and in line with the Declaration of Helsinki, including amendments (1996) and Federal Law No. 86-fz On Medicines. The study protocol (No. 0001 EIPVE version 2 dated 03/19/2010, phase III) and other documents requiring preliminary review were approved by the Ethics Committee of the Ministry of Health of the Russian Federation and the local ethics committee of the municipal institution “Children's Hospital No. 11.” Children of either sex aged 1–15 years were enrolled in the study.

Written informed consent for study participation was obtained from the parents/legal representatives of all subjects prior to their involvement in any study procedures. Inclusion criteria were as follows:

  Healthy children of both genders
  Aged 1–15 years (from their 1^st^ birthday to the day before their 16^th^ birthday)
  Who had not contacted TBEV
  Who had not received an earlier vaccination against any flavivirus infection
  Who did not receive systemic therapy at the time of the study
  Who did not have any limitations or contraindications according to the instructions for vaccine use

The immunization was performed for 1 month. All subjects eligible for inclusion in the present study received two doses of either pediatric TBE vaccine on the respective days of the applicable immunization schedule, i.e., the interval between vaccine doses was 30 days (conventional schedule) or 14 days (rapid schedule). The Tick-E-Vac was administered by intramuscular injection in the M. deltoideus twice with the intervals described above. FSME-IMMUN Junior was administered in the M. deltoideus for children over 18 months, but in the M. vastus lateralis for children younger than 18 months.

### 2.3. Reactogenicity

Following vaccination, subjects were observed for at least 30 min as a precautionary measure in case of immediate postvaccination reactions. The following adverse reactions were recorded by doctors daily for 7 days after each injection: (1) local reactions—itching, burning sensation, local pain (at the injection site), skin reactions (redness, erythema), swelling or tightening at the injection site, swelling of axillary lymph node on the injection side; (2) systemic reactions—fever (measured 2 times a day), sleep disturbances (insomnia) and restlessness in children aged 1–4 years, and a feeling of physical discomfort: chills, nausea, myalgia, arthralgia, vomiting, anorexia, headache, fatigue, anxiety, dizziness, and unsteady gait. Body temperature was measured rectally in children aged <3 years and by axillary thermometer in children aged ≥ 3 years, as well as in adolescents. Any fever that occurred in temporal association with vaccination (i.e., within 7 days) was included in the analysis.

Furthermore, the incidence and severity of unexpected adverse events were reported after the first injection and within 30 days following the second injection.

The process was considered to be asymptomatic if no reactions were observed in the recipient and the body temperature remained within the normal range.

A scoring system was applied to assess the severity of the objective and subjective parameters. Observed adverse reactions (i.e., systemic and local reactions) were divided into the following categories:

1. Mild reactions or body temperature of up to 37.0 °C
2. Moderate reactions or body temperature of 37.1–37.5°C
3. Severe reactions or body temperature of 37.6 °C or higher

### 2.4. Serum Samples

Blood samples were drawn on the following days: day 0 before the first vaccine injection and day 30 after the second vaccine injection according to the conventional schedule and days 14 and 30 if the rapid schedule was used. Collected serum samples were frozen and later stored at temperatures of −20 ± 1°С until a plaque reduction neutralization test was performed. Sera on day 0 were screened by ELISA using commercial kit (VectoTBE-IgG, D-1156 Vector-Best, Novosibirsk, Russia) [11]. In brief, TBEV antibody titer in the serum was assessed according to a calibration curve of the dependence of the optical density on the IgG concentration (U/ml). IgG concentration of 100 U/ml corresponds to a titer of 1 : 100, 200 U/ml to a titer of 1 : 200, etc. And in this study, we describe the results for the primarily seronegative recipients according to the ELISA assay.

### 2.5. 50% Plaque Reduction Neutralization Test (PRNT50)

The PRNT50 analysis was conducted using the Sofjin strain of TBEV (GenBank KC806252) and pig embryo kidney cells (PEK) as previously described [9].

### 2.6. Statistical Analysis

Statistical analysis was performed by standard methods of variance statistic in Microcal Origin 8.0. Since the number of subjects in the groups was relatively small and the obtained data were not normally distributed, the significance of the differences between variables was determined using the Mann–Whitney test (MWT) and the chi-square test (CST). A *p* value of 0.05 was considered significant.

## 3. Results

### 3.1. Demographic and Anthropometric Data of the Recipients

In this study, we analyzed the data only for those subjects of the clinical study for whom full local and systemic reaction data were available and for whom serum samples were collected before and after vaccination and could be tested by PRNT50. Two hundred and twelve children who were eligible for the present study and found to be seronegative based on ELISA results were randomized into two groups and received one of the two vaccines. Thus, 152 participants would be vaccinated according to the conventional schedule (CS) and 60 according to the rapid schedule (RS); however, 49 subjects refused to participate in the study before or after the second injection and were excluded from further study.

The participant's serum samples collected on day 0 before the first vaccine injection were tested for the presence of antiviral antibodies by PRNT50, and TBEV-neutralizing antibodies (nAbs) at a titer ≥1 : 10 were observed in 17 (11.6%) children aged 1.3–15 years. Thereafter, the data obtained for this group of recipients were studied separately.

Of the 146 recipients who did not possess TBEV nAbs at baseline, 76 children were vaccinated with Tick-E-Vac (including 49 children—according to CS and 27—according to RS) and 70 children received FSME-IMMUN Junior (42 according to CS and 28 children according to RS).  Table 1 summarizes the demographic and anthropological characteristics of the study participants, all of whom were Caucasian. As 49 (23.1%) subjects were excluded in the study after randomization, groups receiving Tick-E-Vac or FSME-IMMUN Junior according to the conventional schedule differed slightly in anthropometric features.

### 3.2. Reactogenicity Profile of “Tick-E-Vac” and “FSME-IMMUN Junior”

The adverse reactions following TBE immunization were recorded for both vaccines. All subjects who developed local or systemic signs and symptoms were examined by medical practitioners, and no criteria for the severe unexpected adverse events were found. All reactions resolved without any intervention. In total, local and systemic reactions were registered in 32 (21.9%) children after the first injection and in 15 (10.3%) children after the second one (Table 2). For comparison, 23 (15.1%) cases of adverse reactions were seen in children who received Tick-E-Vac and 24 (17.1%) cases of adverse reactions in children who received FSME-IMMUN Junior. Differences in the incidence of adverse reactions between the vaccines were statistically insignificant (CST).

Adverse reactions after the first and second immunizations were reported in 23 (15.8%) recipients after vaccination according to CS and in 12 (8.2%) children after vaccination according to RS, corresponding to 24% of all cases of vaccine administration. The reactions following both injections were identified in 18 girls and 17 boys aged 1.1–15 years and were often observed in the same subject.

Overall, 72 reactions were registered, including 28 local reactions (such as itching, burning sensation, pain, swelling, and hyperemia at the injection site) and 44 systemic reactions (such as bodily discomfort, fever, and sleep disturbance). In total, 48 reactions occurred after the first injection and 24 reactions occurred after the second injection. In general, either local or systemic reactions were reported in children receiving either the first or the second dose of study vaccines; the number of recipients who experienced reactions of both types was significantly lower. The following reactions were reported most frequently: a feeling of physical discomfort in 27.8%; itching, burning sensation, and local pain at the injection site in 22.2%; sleep disturbance in 18.1%; swelling at the injection site and redness in 16.7%; and fever in 15.2%. The characteristics of the reactions observed in the recipients are presented in Table 3. Differences related to the incidence, severity, and duration of local and systemic reactions between the groups receiving either Tick-E-Vac or FSME-IMMUN Junior were not statistically significant (CST).

We assessed the correlation between groups regarding ages of children and number of children who developed adverse reactions following first or second vaccine injection according to both a conventional or rapid schedule. The correlation coefficients values ranged from −0.1 to 0.2, which were applicable for both vaccines. It was also determined that number of children who developed adverse reactions in the age groups 1–4 (adverse reactions in group (28.1%)), 5–11 (16.2%), and 12–15 (42.9%) years were not significantly different from each other (CST).

### 3.3. Immunogenicity of Tick-E-Vac or FSME-IMMUN Junior

The PRNT50 test with the application of the titration method of the recipient's serum and TBEV strain Sofjin was used to evaluate the immunogenicity of the vaccines (Table 4). For seropositive children, the range of nAbs titers was from 1 : 10 to 1 : 2000. Since the obtained data were not normally distributed, the Mann–Whitney test (MWT) and the chi-square test (CST) were used to perform statistical data analysis.

In the 30 days after the second injection, seroprotection rates (SRs) in the subjects given either vaccine according to the conventional or rapid schedule were over 95%. There were no statistically significant differences identified in the SRs (CST) and nAbs titers (MWT) between groups of children vaccinated with either Tick-E-Vac or FSME-IMMUN Junior. Differences found in the SRs and nAbs titers were not statistically significant across the groups of children who had received two doses according to conventional or rapid vaccination schedules, and there were no significant differences in geometric mean titer of TBEV nAbs (GMTA) between the groups.

However, 14 days after the second dose was administered, the SRs determined in children vaccinated according to the rapid schedule with Tick-E-Vac or FSME-IMMUN Junior were only 63% and 50%, respectively. In this case, GMTA was 1 : 40 for seropositive children who received Tick-E-Vac and 1 : 60 for those who received FSME-IMMUN Junior. The difference between nAbs titers measured on 14^th^ and those measured on the 30^th^ day were statistically significant for both vaccines (*p*=0.001, MWT).

We assessed the correlation between groups regarding nAbs titers and ages of the total group of subjects vaccinated with either vaccine according to both a conventional or rapid schedule. The correlation coefficient values ranged from −0.2 to 0.2, which were applicable for both vaccines and for all time intervals at which the serum samples were analyzed. It was also determined that the SRs and nAbs titers (CST and MWT, accordingly) in children in the age groups 1–4 (64 children), 5–11(68 children), and 12–15 (14 children) years were not significantly different from each other.

### 3.4. Analysis of the Relationship between the Immunogenicity and Reactogenicity of the TBE Vaccines

To examine the relationship between reactogenicity and immunogenicity of vaccination against TBE after two doses according to the conventional or rapid schedule, we compared SRs, nAbs titers, and GMTA in two combined groups. The first group consisted of subjects who developed adverse reactions after receiving either vaccine, and the second group included subjects who had no reported adverse reactions (Table 5). The differences in the SRs and nAbs titers between the two groups were statistically insignificant on day 30 after the second dose was administered. However, for the rapid vaccination schedule on day 14 following the administration of the second dose, a comparison between sera from subjects who had no reactions and those who developed reactions revealed differences in the nAbs titers (*p*=0.00752, MWT). GMTA was 2.3 log_10_ (1 : 182) in the group of subjects who developed adverse reactions, but only 1.5 log_10_ (1 : 32) in the group of subjects who had no reactions.

### 3.5. Reactogenicity and Immunogenicity of Tick-E-Vac or FSME-IMMUN Junior in Participants with nAbs to TBEV before Vaccination

In this group, TBEV nAbs titers ranged from 1.0 to 3.3log_10_ before the first dose of vaccine was given. The GMTA 1.6log_10_ values were determined in the serum samples collected from 17 children (11.6%) (7 girls and 10 boys) aged 1.3 to 15 years.

Local and systemic reactions after the first vaccine injection were observed in 5 children (29.4%) in this group, and 3 (17.6%) of them developed the reactions after the second one. At the same time, the differences between the incidences of adverse reactions reported in this group and in the group of subjects who were seronegative at baseline (21.9% after the first injection and 10.3% after the second, Table 2) did not reach statistical significance. The most frequently reported reactions were as follows: itching, burning sensation, and local pain at the injection site—17.6%; sleep disturbance—17.6%; a feeling of bodily physical discomfort—11.8%; fever—5.9%; and swelling at the injection site and redness—5.9%. All reported reactions were mild, occurred within 1–3 days after vaccination, and lasted no more than 3 days.

The administration of either vaccine according to any schedule led to an increased titer of nAbs against TBEV, and the GMTA identified in this initially seropositive group of subjects was 2.2 log_10_ (1 : 158). On day 30 after the second injection, no statistically significant differences in immunogenicity (SRs and nAbs titers) were identified in the group who were either seronegative or seropositive at baseline.

## 4. Discussion

In the Russian Federation, a licensed domestic vaccine intended for the immunization of young children (aged 1 to 3 years) was not available until 2012. For this reason, imported vaccines based on the European strains of TBEV were used for this population. Data regarding the reactogenicity and immunogenicity of such vaccines used in areas in which Eur subtypes of TBEV circulate have been previously published [7].

A new liquid adsorbed TBE vaccine (Tick-E-Vac) based on the FE strain Sofjin was developed in Chumakov Federal Scientific Center. The vaccine contains purified formaldehyde-inactivated virions, adsorbed on aluminum hydroxide. The vaccine contains no formaldehyde, antibiotics, and preservatives. There are two forms of the vaccine: for persons 16 years old and up in a dose 0.5 ml and for children and adolescents at a dose 0.25 ml. The primary vaccination course for this vaccine consists of two intramuscular injections given according to the conventional or rapid schedule. The interval between injections is from 1 to 7 months (conventional schedule) or 14 days (rapid schedule). The vaccination course could be performed year-round including epidemic season, but not later than 2 weeks before time of visit to TBE natural focus. First revaccination is performed in 1 year after completion of primary vaccination course; subsequent booster vaccinations are carried out every 3 years. Tick-E-Vac can also be used for immunization of blood donors.

The primary course of immunization against TBE should induce antiviral antibodies in protective titers of at least 95% in vaccinated subjects, i.e., provide sufficient protection to allow patients to live in TBE-endemic areas. The primary vaccination course for all TBE vaccines produced in Russia, including the Tick-E-Vac, consists of only two injections followed by revaccinations. Although FSME-IMMUN Junior primary vaccination consists of three injections, the vaccine provides acceptable immunogenicity even by a double immunization schedule [7]. Accordingly, when choosing a vaccine for comparison in the clinical study involving Tick-E-Vac and having only two injections as immunization schedule, FSME-IMMUN Junior, which is known for its low adverse event rates and high immunogenicity was chosen as a comparison drug.

Evaluations of the reactogenicity and immunogenicity of whole virion, inactivated, and concentrated vaccines against TBE have been conducted repeatedly; however, the majority of these studies analyzed collected immunization data corresponding to the conventional vaccination schedule [12–14] or the rapid schedule [15–17]. Data from comparative studies of reactogenicity and immunogenicity of different TBE vaccines using different immunization schedules in a single experiment were rather limited [18].

In this study, we evaluated and compared the reactogenicity profiles and effectiveness of the pediatric version of two vaccines: Tick-E-Vac and FSME-IMMUN Junior. Tick-E-Vac is based on the FE subtype of TBEV and FSME-IMMUN Junior is based on Eur subtype; thus, the TBEV strains used to produce the vaccines differ from strains of the Sib subtype of TBEV which is currently dominating in the Sverdlovsk region [10] in which this study was performed.

As shown previously, the vaccines based on the FE strain Sofjin or Eur strain Neudoerfl of TBEV are characterized by a pronounced immunogenicity based on the antiviral antibody titers, as measured by the enzyme-linked immune sorbent assay (ELISA) [11]. However, data obtained using only the ELISA method do not allow the evaluation of the full degree of protection of the recipients [19, 20].

According to the Russian Ethical Guidelines for clinical trials, individuals living in TBE-endemic areas usually participate in clinical studies with TBE vaccines. A number of works have shown that the level of naturally acquired immunity against TBEV in such regions can account for more than 20% [15, 20]. In the course of the study, we also identified a significant number of children between the ages of 1.3 and 15 who were TBE seropositive based on the PRNT50 results (11.6%, GMTA—1.6 log_10_), but who either did not have a history of TBE infection, had not been vaccinated against TBE, or were seronegative according to the ELISA results. The data on the reactogenicity and immunogenicity registered in these subjects can be significantly distorted by a high level of naturally acquired immunity [21]. Therefore, in the current work, we focused on the analysis of the data obtained from participants who were seronegative before immunization, according to the results obtained by both methods described above (PRNT50 and ELISA). Children were screened by ELISA using VectoTBE-IgG kit. This kit is designed to determine specific antibodies to various subtypes of TBEV and is usually used to assess the immunogenicity of TBE vaccines in Russia [11, 12, 22].

The analysis of reactogenicity of Tick-E-Vac and FSME-IMMUN Junior showed that local and systemic reactions reported in children were mild or moderate. These reactions usually occurred 1‐2 days after immunization and lasted no more than 3‐4 days. The incidence of these reactions for either vaccine was statistically lower after the second dose was given. Although groups vaccinated with Tick-E-Vac or FSME-IMMUN Junior differed in some anthropometric features (Table 1), there were no statistically significant differences in the reactogenicity of the vaccines. The reactogenicity did not correlate with the gender or age of the subjects either.

According to the manufacturer's data, the protein E content is 0.45 ± 0.05 *μ*g per Tick-E-Vac dose and 1.19 *μ*g per dose of FSME-IMMUN Junior. There were no differences in the reactogenicity profiles relative to the amount of protein E per vaccine dose, although one should keep in mind that the manufacturers use different methods for the measurement of protein E.

The PRNT50 method was applied to evaluate immunogenicity using the Sofjin strain of TBEV as the challenge virus. It could be expected to influence the value of nAbs titer in sera collected from subjects immunized with FSME-IMMUN Junior and resulted in lower nAbs titers than the ones for Tick-E-Vac immunized children. But, any statistically significant differences in the immunogenicity of the studied vaccines were not demonstrated. These data are in good agreement with the data for protective efficacy TBE vaccines based on Eur or FE strains against various TBEV strains in vivo and in vitro [23–26]. It was demonstrated that GMTA was at least 2.4 log10 (1 : 250) in the 30 days after the second dose of either vaccine, suggesting that a pronounced immune response is formed and that a protective titer of antiviral nAbs exists. We calculated SR as the percentage of recipients with nAbs titers greater than 1 : 10, values that are usually taken as protective titers against TBE [7]. On day 30 after the second dose was administered, the seroprotection rates were 100% for Tick- E-Vac and more than 95% for FSME-IMMUN Junior. These values did not correlate with the applied immunization schedule. Although groups vaccinated with Tick-E-Vac or FSME-IMMUN Junior differed in some anthropometric features, the difference did not affect immunogenicity.

It should be noted that seroprotection rates were significantly lower on day 14 after the administration of the second dose according to the rapid vaccination schedule, ranging from 50% to 63%, regardless of the vaccine used. Thus, these results should be taken into account when vaccinating children under 16 years old, as they reach the maximum level of seroprotection later than adults, in whom SRs of at least 90% are registered in two weeks after receiving the second dose [27]. Since the probability of previous interactions with TBEV in adults is higher than in children [20], these differences may be associated with higher levels of immunological memory in adults living in TBE-endemic areas than in children living in the same areas, even if recipients are initially seronegative according to data of ELISA and PRNT50.

As for conventional and rapid primary immunization schedules, we did not reveal statistically significant differences in SRs and in antiviral nAbs titers in the 30 days after the second dose was administered, allowing us to begin discussion with respect to transitioning to a universal (single) schedule for primary vaccination against TBE, corresponding to the 0- to 14-day schedule. However, it is necessary to assess long-term immune responses in vaccinated individuals and determine whether a solid immunological memory has built up. Likewise, there was no correlation between the immunogenicity profile and the children's gender and age.

According to Leonova et al., there was a possible correlation between high titers of antibodies against TBEV in subjects who received the vaccine and the incidence and severity of local and systemic reactions due to TBE vaccination reported [21]. Our study demonstrated that SRs, determined at different time intervals after the second dose was administered, did not differ between the combined groups of subjects, who developed adverse reactions after injection with either vaccine or those who had no reactions (Table 5). At the same time, a statistically significant difference between these groups in the antiviral nAbs titers, registered on day 14 after the second dose was given according to the rapid vaccination schedule, were identified, which may indicate a faster increase in nAbs titer in recipients who developed local or systemic reactions. However, it should be noted that the compared groups substantially differed in the number of participants.

We also evaluated the reactogenicity and immunogenicity in subjects who initially possessed antiviral nAbs titers. Overall, 72 local and systemic reactions (9.9% of the total number) were registered in the baseline seronegative recipients, and 10 reactions (5.9%) were registered in the baseline seropositive subjects. There were no statistically significant differences between these two groups of recipients with regard to reactogenicity. In the 30 days after the second injection, the differences in SRs and nAbs titers were not statistically significant either.

Thus, the obtained data on the incidence, severity, and duration of local and systemic reactions in children, who for the first time received Tick-E-Vac or FSME-IMMUN Junior according to the conventional or rapid vaccination schedule, suggest acceptable reactogenicity for both vaccines studied. High rates of immunogenicity of the TBE vaccines studied were demonstrated as well. At the same time, any significant differences in the reactogenicity or immunogenicity of the studied vaccines based on strains of the FE or Eur TBEV subtypes have not been determined.

## 5. Conclusions

The obtained data suggest acceptable reactogenicity and high immunogenicity of the pediatric form of Tick-E-Vac and FSME-IMMUN Junior. The observed adverse reactions were mild or moderate for both vaccines and were not associated with the gender or age of the recipients. Under conventional and rapid vaccination schedules, in the thirty days after the second injection, the two- dose primary immunization induced protective titers of TBEV neutralizing antibodies in protective titers in at least 95% of vaccinated subjects. At the same time, any differences in the reactogenicity or immunogenicity of the studied vaccines based on strains of FE or Eur TBEV subtypes have not been demonstrated in children living in the region in which strains of the Sib subtype of TBEV are dominating.

## Figures and Tables

**Table 1:** Demographic and anthropological characteristics of the study groups.

Characteristics	Parameter	Conventional schedule		Rapid schedule	
		Tick-E-Vac	FSME-IMMUN Junior	Tick-E-Vac	FSME-IMMUN Junior
Number of subjects	Total number (*N* = 146)	49	42	27	28

Gender	Female/male	23/26	19/23	11/16	11/17

Age (years)	Mean	7.4^a^	4.7^a^	4.5	4.0
	Median	7.0	3.0	5.0	4.0
	Range	1–15	1.1–15	3–6	1.5–9

Height (сm)	Mean	124.2^b^	107.1^b^	109.7	103.9
	Median	122.0	100	110.0	105.5
	Range	77–176	75–165	94–126	81–132

Weight (kg)	Mean	28.2^c^	20.5^c^	17.9	16.6
	Median	24.6	15.6	18.0	16.6
	Range	8.2–60	10.2–53	14.1–24.5	9.9–24

^a,b,c^Statistically significant differences of age, height, and weight between two groups of subjects who received Tick-E-Vac or FSME Immune injection according conventional schedule (MWT).

**Table 2:** Adverse reactions after vaccination with Tick-E-Vac or FSME-IMMUN Junior.

Schedule	Tick-E-Vac			FSME-IMMUN Junior		
	*N*	*n*	%	*N*	*n*	%
	After the first dose administered					
Conventional	49	10	20.4	42	11	26.2
Rapid	27	6	22.2	28	5	17.9
In total for the two schedules	76	16^a^	21.1	70	16^b^	22.9

	After the second dose administered					
Conventional	49	6	12.2	42	7	16.7
Rapid	27	1	3.7	28	1	3.6
In total for the two schedules	76	7^a^	9.2	70	8^b^	11.4

	In total after both doses of vaccine administered					
Conventional	49	11	22.4	42	12	28.6
Rapid	27	7	25.9	28	5	17.9
In total for the two schedules	76	18	23.7	70	17	24.3

*N*—total number of subjects; *n*—number of subjects who developed the adverse reactions. ^a,b^Statistically significant differences of reverse reactions after the first injection and after the second one (CST).

**Table 3:** Characteristics of local and systemic reactions in children vaccinated with Tick-E-Vac and FSME-IMMUN Junior.

Vaccination	Reaction	Vaccine (T, I)	Number of subjects (%)	Severity^*∗*^	Onset interval (days)	Duration (days)
Conventional schedule						
1	Local	T	7/14.3	1	1	1–2
		I	8/19.0	1–2	1–2	1–3
	Systemic	T	6/12.2	1–2	1	1–3
		I	10/23.8	1–2	1–2	1–3

2	Local	T	3/6.1	1–2	1–3	1
		I	2/4.8	1	1	2
	Systemic	T	5/10.2	1–3	1–3	3 or more
		I	11/26.2	1–2	1–2	3 or more

Rapid schedule						
1	Local	T	2/7.4	1	1	1
		I	5/17.9	1	1	2
	Systemic	T	6/22.2	1–2	1–2	1–4
		I	4/14.3	1	1	1–2

2	Local	T	—	—	—	—
		I	1/3.6	1	1	3
	Systemic	T	2/7.4	1	2	2
		I	—	—	—	—

T, Tick-E-Vac; I, FSME-IMMUN Junior. ^*∗*^Severity scores (see Materials and Methods).

**Table 4:** Immunogenicity of Tick-E-Vac and FSME-IMMUN Junior.

Schedule	Vaccine	Tick-E-Vac				FSME-IMMUN Junior			
	Blood sampling	*N*	SRs, %	GMTA, log_10_		*N*	SRs, %	GMTA, log_10_	
				Total	Only seropositive			Total	Only seropositive
CS	30 days after administration of the second dose	49	100	**2.5**	**2.5**	42	95.2	**2.4**	**2.5**
				*M* = 2.5 (1.0–3.3)	*M* = 2.5 (1.0–3.3)			*M* = 2.4 (0–3.3)	*M* = 2.5 (1.1–3.3)

RS	14 days after administration of the second dose	27	63	**1.0** ^a^	**1.6** ^b^	28	50	**0.9** ^c^	**1.8** ^d^
				*M* = 1.2 (0–3.3)	*M* = 1.4 (1–3.3)			*M* = 0.5 (0–3.3)	*M* = 1.8 (1–3.3)
	30 days after administration of the second dose	27	100	**3.1** ^a^	**3.1** ^b^	28	96.4	**2.4** ^c^	**2.5** ^d^
				*M* = 3.3 (1.1–3.3)	*M* = 3.3 (1.1–3.3)			*M* = 2.3 (0–3.3)	*M* = 2.3 (1.4–3.3)

CS: conventional schedule; RS: rapid schedule; *N*: total number of subjects in the groups; GMTA: geometric mean titer of TBEV-neutralizing antibodies; M: median; ( ): nAbs titer range; ^a,b,c,d^difference between the nAbs titers measured on day 14 and those measured on day 30 after immunization according to rapid schedule (MWT).

**Table 5:** Relationship between immunogenicity and reactogenicity of the TBE vaccines.

Schedule	Subjects	Number of subjects	Immunogenicity^*∗*^ (after 2 doses)	
			14 days	30 days
Conventional	With reactions	23	nd	95.7/2.2 (1.1–3.3)
	With no reactions	68	nd	98.5/2.6 (1.0–3.3)

Rapid	With reactions	12	66.7/2.3^a^ (1.3–3.3)	100/2.8 (1.6–3.3)
	With no reactions	43	53.5/1.5^a^ (1.0–3.3)	97.7/2.8 (1.1–3.3)

^*∗*^Numerator, SRs; denominator, GMTA. ( ), nAbs titer range; nd, not determined. ^a^Difference in nAbs titers (MWT).

## Data Availability

Clinical trials data are published in [11] and presented in this study. Raw protocols are available upon request.

## Supplementary Materials

Supplementary Materials The supplementary file is the English version of the official information for the user of Tick-E-Vac (instruction of Tick-E-Vac application in Russian). The last version of the instruction was approved on 3/16/2017. Instruction of Tick-E-Vac application includes the following: composition, immunological and biological properties, indication, contraindications, safety, application and dosage, the vaccination schedules, possible adverse reactions and vaccine overdose, pediatric and adult form description, and storage and transportation conditions.
