# Supplementary material for: Comparison of the Immunogenicity and Safety of Two Pediatric TBE Vaccines Based on the Far Eastern and European Virus Subtypes
Source: Adv Virol. 2019 Dec 24;2019:5323428. doi: 10.1155/2019/5323428 (PMC6942698; doi:10.1155/2019/5323428)
Supplement: Supplementary Materials — The supplementary file is the English version of the official information for the user of Tick-E-Vac (instruction of Tick-E-Vac application in Russian). The last version of the instruction was approved on 3/16/2017. Instruction of Tick-E-Vac application includes the following: composition, immunological and biological properties, indication, contraindications, safety, application and dosage, the vaccination schedules, possible adverse reactions and vaccine overdose, pediatric and adult form description, and storage and transportation conditions. [file 5323428.f1.pdf]

MINISTRY OF HEALTH OF THE RUSSIAN FEDERATION
INSTRUCTION
of medical application of the medicinal product
Tick-E-Vac
(Tick-borne encephalitis vaccine cultural purified concentrated inactivated absorbed)

Registration number:
Commercial name: Tick-E-Vac (tick-borne encephalitis vaccine cultural purified concentrated inactivated absorbed)
Common name: Encephalitis vaccine
Route of Administration: Suspension for intramuscular injection
Composition
The single immunizing dose for persons over the age of 16 (0.5 ml) contains:
Active ingredient: inactivated antigen of the virus КЭ – titer no less than 1:128
Excipients: human albumin (solution for injection* 10% or 20%) – 0.25 mg, sucrose – 30 mg, aluminum hydroxide – 0.4 mg, buffer system salts: sodium chloride – 3.8 mg, trometamol – 0.06 mg.
The single immunizing dose for children from 1 to 16 years old (0.25 ml) contains:
Active ingredient: inactivated antigen of the virus КЭ – titer no less than 1:128
Excipients: human albumin (solution for injection* 10% or 20%) – 0.125 mg, sucrose – 15 mg, aluminum hydroxide – 0.2 mg, buffer system salts: sodium chloride – 1.9 mg, trometamol – 0.03 mg.
*Solutions for human albumin injection contain (except human albumin) sodium caprylate and sodium chloride.
The vaccine contains no formaldehyde, antibiotics and preservatives.
Description
Homogenous suspension of white colour, includes no foreign particles.
Pharmacological group
Medical immune-and-biological preparations – vaccine
ATC-Code – J07BA01
Immune-and-biological properties
The vaccine is purified concentrated suspension of the tick-borne encephalitis (TBE) virus (strain «Sofjin»), inactivated with formaldehyde, obtained through reproduction in primary chicken embryo cell culture and absorbed with aluminum hydroxide. The vaccine stimulates the induction of cellular and humoral immunity against TBE virus. The virus-neutralizing antibodies were detected to appear in 90% of vaccinees after two subsequent injections of the vaccine (vaccination course).
Indication
Specific prophylaxis of TBE in children from 1 to 16 years old – at a dose of 0.25 ml, and in persons over 16 years old – at a dose of 0.5 ml;
vaccination of blood donors to obtain specific immunoglobulin.
Vaccination contingents:
1. Resident population inhabiting the territories, enzootic for TBE.
2. Persons visiting the territories, enzootic for TBE, and employed in the following kind of works:
– Agricultural, hydromeliorative, construction, earth excavation or replacement, procurement of agricultural products, hunting, geological surveying, field expedition works, deratization, disinfestation;
– Lumbering, clearance and rehabilitation of forests, country parks and recreation areas.
3. Occasional visiting the territories, enzootic in the respect of TBE, with purpose of rest, tourism, working in truck patches or garden-plots.

4. All kinds of works with live cultures of TBE etiological agent.

Contraindications

1. Acute infectious and non-infectious diseases, chronic diseases in the exacerbation phase – the vaccination should be performed no earlier than in one month after the recovery (remission).
2. Severe allergic reactions in anamnesis; bronchial asthma; autoimmune diseases.
3. Allergic reactions to vaccine components in anamnesis.
4. Heavy reaction (increase of body temperature over 40 °C, swelling and hyperemia over 8 cm in diameter) or complication after previously inoculated dose of the preparation.
5. Children under the age of 1 year.

While immunizing blood-donors the contraindications listed above as well as contraindications related to election of donors should be taken into consideration.

If a person to be vaccinated is having a disease not included to the above list, the vaccination is carried out only by permission of local medical service given in accordance with the patient's state of health and the risk of catching TBE. In order to reveal contraindications the physician (medical attendant) has to interview each the vaccinee and carry out his/her medical observation with thermometry at the day of vaccination.

Safety precautions

Warning! *Injection of preparation by intravenous administration is forbidden.*

Special instructions

The vaccination should be carried out under strict aseptic and antiseptic conditions.

All vaccination stations should be supplied with anti-shock and anti-allergic remedies.

Inspect each ampoule visually before opening.

The preparation contained in damaged or improperly labeled ampoules, containing foreign particles or large conglomerates, or expired, or stored and transported under improper temperature conditions is forbidden for use.

Immediately before the injection, shake an ampoule with the vaccine – the content of the ampoule should form a homogenous suspension. The vaccine is injected immediately after opening of the ampoule with a syringe intramuscularly into deltoid muscle of the shoulder.

Each immunization has to be registered in a special form where the preparation name, vaccination dates, dose, lot number, vaccine manufacturer, adverse reactions (if any) are indicated.

Pregnancy and lactation

No clinical trials with Tick-E-Vac vaccine have been conducted on pregnant or lactating women.

Only after careful identification of potential risk of TBE-virus infection, vaccination of pregnant women can be performed.

Vaccination of lactating women can be performed in two weeks after delivery.

Application and dosage

The vaccine is injected with a syringe intramuscularly into deltoid muscle of the shoulder.

1. Prophylactic vaccination.

1.1. Routine immunization

Primary immunization course consists of two intramuscular injections, single dose each, done at a 1-7 month interval. One vaccination dose is: for persons over the age of 16 – 0.5 ml; for children from 1 to 16 years old – 0.25 ml.

The vaccination course could be performed year-round including epidemic season, but not later than 2 weeks before time of visit to TBE natural focus.

The optimal interval between the first and the second injections is 5-7 months (autumn-spring).

1.2. Emergency vaccination

Emergency vaccination can be performed by epidemic indications. In such a case, the vaccine is administered twice at a 2-weeks interval: for persons over the age of 16 – 0.5 ml, and for children from 1 to 16 years old – 0.25 ml. The vaccination could be performed not later than 2 weeks before time of visit to TBE natural focus.
First revaccination is performed in 1 year after completion of primary vaccination course with the following vaccination dose: for persons over the age of 16 – 0.5 ml; for children from 1 to 16 years old – 0.25 ml.
Subsequent revaccinations are carried out as a single shot every 3 years in accordance with the age of a patient.

General vaccination schedule

**Vaccination type**	**Primary vaccination**		**First revaccination**	**Subsequent revaccination**
	**First**	**Second**		
Routine	vaccination day 0	In 1-7 months after the first vaccination	In 12 months after the second vaccination	Every 3 years
Emergency		In 2 weeks after the first vaccination		
Single dose for persons over the age of 16	0.5 ml	0.5 ml	0.5 ml	0.5 ml
Single dose for children from 1 to 16 years old	0.25 ml	0.25 ml	0.25 ml	0.25 ml

2. Vaccination of blood donors
Primary course consists of two 0.5 ml injections at an interval of 5-7 months, or of three 0.5 ml injections at an interval of 3-5 weeks between them. The first of two above-mentioned schedules provides better immunizing effect. Revaccination is performed with a dose of 0.5 ml 6-12 months after completion of the primary vaccination course. The first blood taking from the immunized donors is carried out 14-30 days after the vaccination.

Possible adverse reactions

After inoculation of the vaccine some local or generalized reactions may be observed in rare cases.
The evaluation criteria of frequency of adverse reactions occurrence: very frequently (> 1/10), frequently (≥ 1/100 to <1/10), infrequently (≥ 1/1000 to <1/100), rarely (≥ 1/10000 to <1/1000), very rarely (< 1/10000).
For persons over the age of 16:
Local reactions:
Frequently: redness, swelling, painfulness in injection site.
Very rarely: infiltrate development, insignificant enlargement of local lymph nodes.
Local reactions may develop within the first 2 days after inoculation. Duration of the local reactions does not exceed 3 days.
General reactions:
Frequently: general malaise, headache, nausea, temperature increase up to 37.5 °C.
Infrequently: temperature increase from 37.5 °C up to 38.5 °C.
Rarely: temperature increase higher that 38.5 °C.

General reactions may develop within the first 2 days after inoculation; duration of the general reactions does not exceed 2 days.
For children from 1 to 16 years old:
Local reactions:
Frequently: redness, swelling, painfulness in injection site.
Very rarely: infiltrate development, insignificant enlargement of local lymph nodes.
Local reactions may develop within the first 2 days after inoculation. Duration of the local reactions does not exceed 3 days.
General reactions:
Very frequently: temperature increase up to 37.5 °C.
Frequently: general malaise, headache, nausea, temperature increase up to 38.5 °C.
Rarely: temperature increase higher that 38.5 °C.
Local reactions may develop within the first 3 days after inoculation. Duration of the local reactions does not exceed 3 days.
General and local adverse reactions more often occur after the first inoculation.
As an exclusion, inoculation may cause an allergy reactions of immediate type, therefore all vaccinees have to be under medical observation within 30 minutes after the immunization. All vaccination stations should be supplied with anti-shock and anti-allergic remedies.

Vaccine overdose

No cases of vaccine overdose were identified. Potential risk of overdose by the vaccine is not investigated.

Taking other medicines

The vaccination against TBE can be performed simultaneously (on the same day) with inoculation of another inactivated vaccines, indicated in National Immunization Schedule and Immunization Schedule by Epidemic Indications (except preventive immunization with rabies vaccine). In other cases the vaccination against TBE should be performed no earlier than one month after the vaccination against any other infection.

Production shape

Suspension for intramuscular injection.
Vaccine in ampoules: 0.5 ml – a single dose for persons over the age of 16, or 0.25 ml – a single dose for children from 1 to 16 years old. A carton box contains 10 ampoules, instruction on application and an ampoule knife (if necessary).

Effects on the ability to drive and use machines

Evident general reactions (temperature increase, headache) are the contraindications for driving and machines using.

Shelf life

Shelf life of the vaccine – 2 years. Do not use the preparation after the expiry date.

Storage conditions

The vaccine is to be stored in accordance with Sanitary Rules СП 3.3.2.3332-16 at the temperature level of 2 to 8 °C. Do not freeze.
Keep out of reach of children.

Transportation conditions

The preparation is to be transported in accordance with Sanitary Rules СП 3.3.2.3332-16 at a temperature of 2 to 8 °C. Transportation at a temperature of 9 to 25 °C within no more than 2 days is allowed.
Do not freeze.

Prescription status

For medical-and-prophylactic and sanitary-and-prophylactic institutions.

Manufacturer

Federal State Budgetary Scientific Institution «Chumakov Federal Scientific Center for Research and Development of Immune-And-Biological Products of Russian Academy of Sciences» (FSBSI «Chumakov FSC R&D IBP RAS»), Russia

Village of Institute of Poliomyelitis, Settlement 'Moskovskiy', Moscow

All claims should be addressed to the Marketing Authorization Holder

FSBSI «Chumakov FSC R&D IBP RAS», Russia

Premises 8, building 1, Village of Institute of Poliomyelitis, Settlement 'Moskovskiy', Moscow, 108819

Tel. (495) 841-90-02, Fax (495) 841-93-21, 549-67-60.

E-mail: sue_polio@chumakovs.ru

First Deputy Director General

FSBSI «Chumakov FSC R&D IBP RAS» A.Yu. Afonin
